# The Dual Role of the β_2_-Adrenoreceptor in the Modulation of IL-17 and IFN-γ Production by T Cells in Multiple Sclerosis

**DOI:** 10.3390/ijms23020668

**Published:** 2022-01-08

**Authors:** Mikhail Melnikov, Vladimir Rogovskii, Anastasiya Sviridova, Anna Lopatina, Mikhail Pashenkov, Alexey Boyko

**Affiliations:** 1 Department of Neuroimmunology, Federal Center of Brain research and Neurotechnology of the Federal Medical-Biological Agency of Russia, 117513 Moscow, Russia; qwer555@mail.ru (V.R.); anastasiya-ana@yandex.ru (A.S.); ank4lopatina@yandex.ru (A.L.); 2 Laboratory of Clinical Immunology, National Research Center Institute of Immunology of the Federal Medical-Biological Agency of Russia, 115478 Moscow, Russia; mvpashenkov@yandex.ru; 3 Department of Neurology, Neurosurgery and Medical Genetics, Pirogov Russian National Research Medical University, 117997 Moscow, Russia; 4 Department of Molecular Pharmacology and Radiobiology, Pirogov Russian National Research Medical University, 117997 Moscow, Russia

**Keywords:** β_2_-adrenoreceptor, norepinephrine, Th1 and Th17 cells, multiple sclerosis

## Abstract

Norepinephrine is a neurotransmitter that also has an immunomodulatory effect and is involved in multiple sclerosis (MS) pathogenesis. This study aimed to clarify the role of the β_2_-adrenoreceptor in the norepinephrine-mediated modulation of interleukin-17 (IL-17) and interferon-γ (IFN-γ) production, which play a critical pathogenetic role in MS. CD4^+^ T cells obtained from twenty-five relapsing-remitting MS patients and sixteen healthy subjects were cultured ex vivo with norepinephrine and/or β_2_-adrenoreceptor antagonist or agonist, followed by a cytokine production analysis using ELISA. Norepinephrine suppressed IL-17 and IFN-γ production by the anti-CD3/anti-CD28-microbead-stimulated CD4^+^ T cells in both groups. Blockade of the β_2_-adrenoreceptor with the specific antagonist ICI 118.551 enhanced norepinephrine-mediated IL-17 suppression but decreased its inhibitory effect on IFN-γ production in MS patients. In contrast, the β_2_-adrenoreceptor agonist formoterol did not influence norepinephrine’s inhibitory effect on cytokine production in both groups. The blockade of the β_2_-adrenoreceptor, even in the absence of exogenous norepinephrine, suppressed IL-17 production but did not influence IFN-γ production in both groups. Conversely, β_2_-adrenoreceptor activation by formoterol decreased IFN-γ production and did not affect IL-17 production in both groups. These data illustrate the inhibitory effect of norepinephrine on IL-17 and IFN-γ production by CD4^+^ T cells in MS. The inhibitory effect of norepinephrine on IFN-γ production by CD4^+^ T cells in MS could be mediated via β_2_-adrenoreceptor activation.

## 1. Introduction

Multiple sclerosis (MS) is the most common demyelinating and neurodegenerative disease of the central nervous system (CNS), which predominantly affects young adults.

The study of neuroimmune interaction is one of the most rapidly developing areas in MS research. Biogenic amines are direct mediators of this interaction. Recent data suggest that biogenic amines can play a significant role in MS pathogenesis using two mechanisms [1]. In particular, the impaired functioning of biogenic amines can cause the development of the neuropsychological symptoms in MS, such as cognitive impairments, fatigue, and mood disorders, which can decrease adherence to therapy, induce stress-related relapses, and, therefore, aggravate the course of MS.

On the other hand, the direct effect of biogenic amines on the immune cells’ function is also possible. It has been shown that the cells of both innate and adaptive immune systems express receptors to biogenic amines [1]. Furthermore, the ability of immune cells to produce catecholamines has been demonstrated, suggesting the possible autoregulation by activating dopaminergic and noradrenergic receptors. Finally, biogenic amines’ involvement in MS pathogenesis is confirmed by the influence of serotoninergic and dopaminergic drugs on experimental encephalomyelitis (EAE) and MS course and the impact of disease-modifying therapeutics (DMT) of MS on the production of catecholamines by the immune cells [2,3,4]. In line with these data, the repurposing of dopaminergic, noradrenergic, and serotoninergic therapeutics as additional pathogenetic treatments in MS is discussed [3,4].

Norepinephrine is one of the key neurotransmitters of the CNS. The role of norepinephrine in MS is multifunctional. Similar to other neurotransmitters, norepinephrine mediates neuropsychological impairments and autonomic dysfunction in MS. The reciprocal interaction between norepinephrine and gut microbiota, which participate in CNS demyelinating disease development has been shown [1]. Much attention has also been paid to the effect of norepinephrine on immune cell function in MS. It has been shown that norepinephrine can affect MS pathogenesis by the modulation of CD4^+^ T cell subsets, including Th1, Th17, and Treg cells [4,5]. According to data in the literature, CD4^+^ T cells express α- and β-adrenergic receptors; however, the subtype of β_2_-adrenergic receptors may have crucial importance in the influence of norepinephrine on CD4^+^ T cells [6,7].

At the same time, the role of the β_2_-adrenoreceptor in norepinephrine-mediated modulation of Th17 cells in MS is unclear. The aim of this study was to clarify the involvement of the β_2_-adrenergic receptor in the modulation of IL-17 and IFN-γ in CD4^+^ T cells in MS.

## 2. Results

### 2.1. The Influence of Norepinephrine on IL-17 and IFN-γ Production by CD4^+^ T Cells and PBMCs

To study IL-17 and IFN-γ production, we activated CD4^+^ T cells and PBMCs with anti-CD3/anti-CD28 microbeads. We found that unstimulated or stimulated cytokine production was not different between the groups (Table 1 and Table 2). The clinical characteristics of MS patients could explain the comparable cytokine secretion. Thus, all MS patients were examined during clinical remission under treatment with glatiramer acetate. In addition, there were no neuropsychological symptoms, such as depression or cognitive impairments in the MS patients.

**Figure 1:**
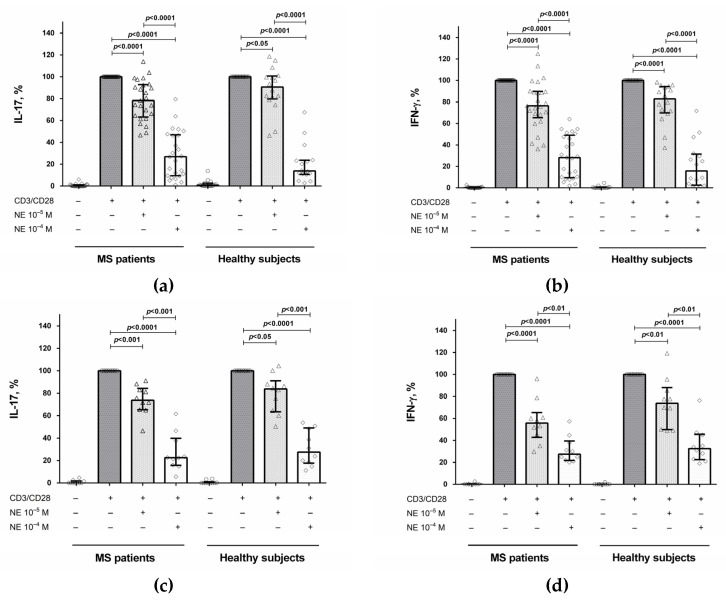
The influence of norepinephrine on IL-17 and IFN-γ production by stimulated CD4^+^ T cells (**a**,**b**) and PBMCs (**c**,**d**) in MS patients and healthy subjects. CD4^+^ T cells or PBMCs (8 × 10^4^ per 200 μL per well), obtained from MS patients or from healthy subjects, were activated with microbeads coated with anti-CD3 and anti-CD28 antibodies with or without norepinephrine at concentrations of 10^−5^ M and 10^−4^ M. After 72 h, the supernatants were collected and submitted to cytokine quantification by ELISA. Data are expressed as the percentage of cytokine production by stimulated cells in the absence of norepinephrine. The boxes in the graphs correspond to the medians and whiskers indicate the 25th and 75th percentiles. The symbols in the graphs correspond to the data from each separately MS patient or healthy subject. The median values of the MS and control groups were compared, and the *p* values are indicated in the figure.

To study the effect of norepinephrine on cytokine production, we stimulated CD4^+^ T cells with anti-CD3/anti-CD28 microbeads in the presence of norepinephrine at various concentrations (10^−6^ M, 10^−5^ M, and 10^−4^ M). It was found that at a concentration of 10^−6^ M, norepinephrine had no effect on cytokine production and proliferative responses (data not shown). At a concentration of 10^−5^ M, norepinephrine reduced IL-17 and IFN-γ production by CD4^+^ T cells in both groups (Figure 1a,b) without affecting proliferative responses (data not shown). At a concentration of 10^−4^ M, norepinephrine further suppressed cytokine production (Figure 1a,b) but also reduced proliferative responses in both groups (data not shown). Therefore, in subsequent experiments, norepinephrine was used at a concentration of 10^−5^ M. In addition, we examined the effect of norepinephrine in some samples of PBMCs. We found that norepinephrine suppressed IL-17 and IFN-γ production by stimulated PBMCs in both groups (Figure 1c,d), which is similar to the influence of norepinephrine on CD4^+^ T cells.

### 2.2. The Role of the β_2_-Adrenergic Receptor in the Modulation of IL-17 and IFN-γ Production by CD4^+^ T Cells and PBMCs

**Figure 2:**
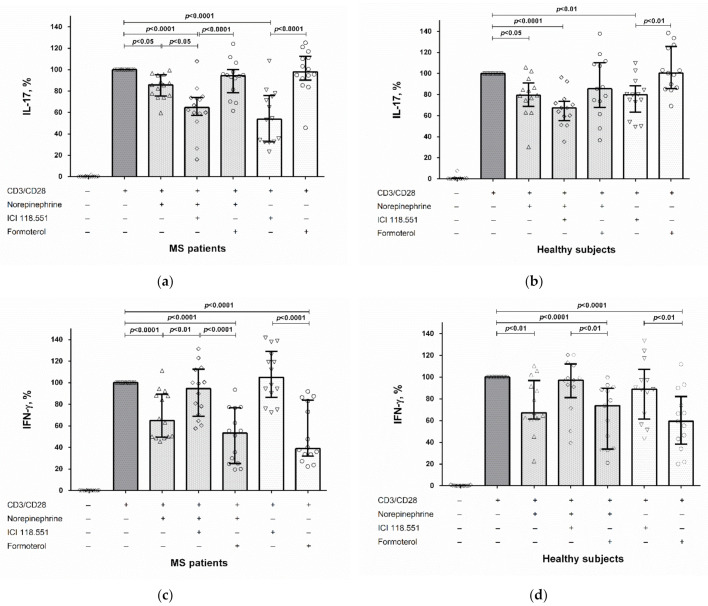
The role of the β_2_-adrenergic receptor in the norepinephrine-mediated suppression of IL-17 (**a**,**b**) and IFN-γ (**c**,**d**) production by stimulated CD4^+^ T cells in MS patients and healthy subjects. CD4^+^ T cells (8 × 10^4^ per 200 μL per well), obtained from MS patients or from healthy subjects, were pre-incubated with an antagonist or an agonist of the β_2_-adrenergic receptor (ICI 118.551 and formoterol, respectively) (at 10^−6^ M), whereafter norepinephrine (at 10^−5^ M) and anti-CD3/anti-CD28 microbeads or anti-CD3/anti-CD28 microbeads (without norepinephrine) were added to the cultures. After 72 h, the supernatants were collected and submitted to cytokine quantification by ELISA. Data are expressed as the percentage of cytokine production by stimulated cells in the absence of norepinephrine and β_2_-adrenergic receptor antagonist or agonist. The boxes in the graphs correspond to the medians and the whiskers indicate to 25th and 75th percentiles. The symbols in the graphs correspond to the data from each separately MS patient or healthy subject. The median values of the MS and control groups were compared, and the *p* values are indicated in the figure.

**Figure 3:**
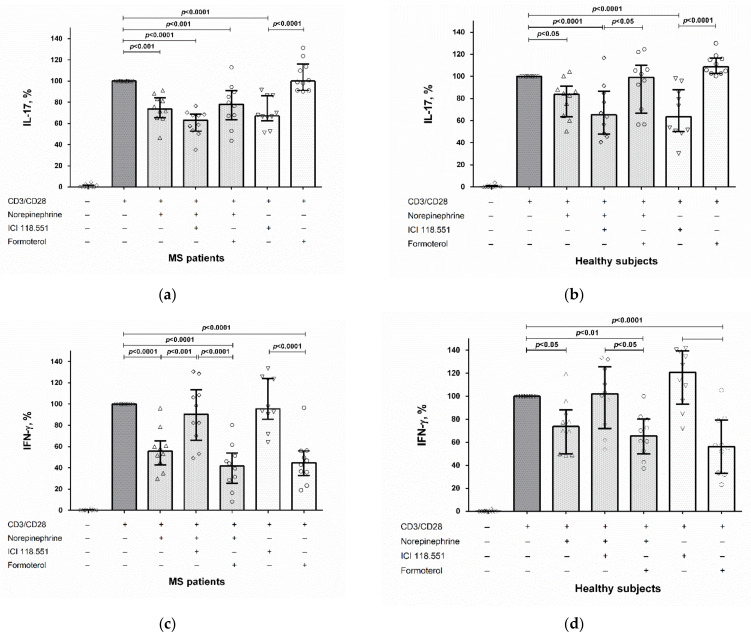
The role of the β_2_-adrenergic receptor in the norepinephrine-mediated suppression of IL-17 (**a**,**b**) and IFN-γ (**c**,**d**) production by stimulated PBMCs in MS patients and healthy subjects. CD4^+^ T cells (8 × 10^4^ per 200 μL per well), obtained from MS patients or from healthy subjects, were pre-incubated with an antagonist or an agonist of the β_2_-adrenergic receptor (ICI 118.551 and formoterol, respectively) (at 10^−6^ M), whereafter norepinephrine (at 10^−5^ M) and anti-CD3/anti-CD28 microbeads or anti-CD3/anti-CD28 microbeads (without norepinephrine) were added to the cultures. After 72 h, the supernatants were collected and submitted to cytokine quantification by ELISA. Data are expressed as the percentage of cytokine production by stimulated cells in the absence of norepinephrine and β_2_-adrenergic receptor antagonist or agonist. The boxes in the graphs correspond to the medians and the whiskers indicate to 25th and 75th percentiles. The symbols in the graphs correspond to the data from each separately MS patient or healthy subject. The median values of the MS and control groups were compared, and the *p* values are indicated in the figure.

Blockade of the β_2_-adrenergic receptor by ICI 118.551 further reduced IL-17 production by CD4^+^ T cells in MS patients but not in healthy subjects (Figure 2a,b). The β_2_-adrenergic receptor antagonist had no effect on the norepinephrine-mediated suppression of IL-17 production by activated PBMCs in both groups (Figure 3a,b). Conversely, blockade of the β_2_-adrenergic receptor reduced the inhibitory effect of norepinephrine on IFN-γ production by stimulated CD4^+^ T cells and PBMCs in MS patients (Figure 2c and Figure 3c). We did not find any influence of the β_2_-adrenergic receptor agonist formoterol on the modulatory effect of norepinephrine on cytokine production by CD4^+^ T cells and PBMCs in both groups (Figure 2 and Figure 3).

Finally, the direct modulatory effects of the β_2_-adrenergic receptor antagonist and agonist on IL-17 and IFN-γ production were observed. The β_2_-adrenergic receptor antagonist, even in the absence of exogenous norepinephrine, suppressed IL-17 production by stimulated CD4^+^ T cells and PBMCs in both groups without affecting IFN-γ production (Figure 2a,b and Figure 3a,b), while the β_2_-adrenergic receptor agonist suppressed IFN-γ production but did not affect IL-17 production in both groups (Figure 2c,d and Figure 3c,d).

## 3. Discussion

Th17 cells are considered to be a crucial factor of neuroinflammation in MS. Almost all pathogenetic MS treatments affect Th17 cell function [8]. The effect of these treatments on immune cells in the CNS still needs to be clarified, suggesting the relevance of the new therapeutic approaches that allow the modulation of the Th17 immune response directly in the CNS [9]. In this regard, the effect of catecholamines on Th17 cells has attracted increasing attention.

The role of norepinephrine and the β_2_-adrenergic receptor in the modulation of Th17 cells was proposed in some previous studies. However, the results of these studies were observed in model animals or in healthy subjects. The present study indicates the ability to modulate Th17 cell function by targeting the β_2_-adrenergic receptor in MS patients. It should be noted that by careful patient selection we have excluded the main known factors that may alter catecholamine functioning, such as depression, cognitive impairments, and therapy with IFN-β. In our group of MS patients, selected according to these criteria, the levels of epinephrine, norepinephrine, and its metabolite 3-methoxy-4-hydroxyphenylglycol (MHPG) in blood plasma were not different from those in healthy donors (Table 3).

Firstly, we have shown that norepinephrine suppresses IL-17 and IFN-γ production by activated CD4^+^ T cells and PBMCs (Figure 1), which corresponds to our previous data and confirms the anti-inflammatory effect of norepinephrine on Th17 cells in MS and EAE [10,11]. It is important to note that in the present study we used a lower concentration of norepinephrine (10^−5^ M) than previously (10^−4^ M) and showed a clear inhibitory effect of norepinephrine on cytokine production without the reduction of cell viability and proliferative response. Although this concentration is still higher than that detected in plasma, norepinephrine concentration in the immediate vicinity of lymphocytes in secondary lymphoid organs can reach 3 mM, and its relatively high concentrations (≥10^−6^ M) are required for the modulation the immune cell function in vitro [12].

Secondly, we found the divergent role of the β_2_-adrenergic receptor in the mediation of norepinephrine’s effect on IFN-γ and IL-17 production by activated CD4^+^ T cells and PBMCs. Thus, the β_2_-adrenergic receptor antagonist ICI 118.551 abolished the norepinephrine-mediated IFN-γ suppression in MS patients (Figure 2c and Figure 3c). The same results were reported by Wahle et al., who showed that the non-selective β-adrenergic receptor antagonist propranolol blocked the inhibitory effect of norepinephrine (at 10^−5^ M) on IFN-γ production by anti-CD3/CD28-activated T cells in rheumatoid arthritis patients and healthy subjects [13]. Conversely, β_2_-adrenergic receptor activation by formoterol had no effect on IFN-γ inhibition by norepinephrine (Figure 2c,d and Figure 3c,d).

In contrast to the effect on IFN-γ, the β_2_-adrenergic receptor antagonist did not affect IL-17 suppression by norepinephrine (Figure 2a,b and Figure 3a,b). Furthermore, the blockade of the β_2_-adrenergic receptor enhanced norepinephrine-mediated IL-17 production by stimulated CD4^+^ T cells in MS patients (Figure 2a). On the contrary, formoterol did not influence the effect of norepinephrine on IL-17 production by CD4^+^ T cells and PBMCs in both groups (Figure 2a,b and Figure 3a,b).

At the same time, the role of the β_2_-adrenergic receptor in the norepinephrine-mediated suppression of IL-17 production is controversial. Liu et al. showed that norepinephrine (10^−5^ M) suppresses Th17 cell function via the β_2_-adrenergic receptor. Thus, ICI 118.551 (10^−6^ M) blocked the inhibitory effect of norepinephrine on IL-17 production by CD4^+^ T cells stimulated with anti-CD3/CD28-antibodies in mice with collagen-induced arthritis [14]. The same results were obtained in the study by Vujnović et al., who reported that propranolol reduces the inhibitory influence of norepinephrine on CD4^+^ IL-17-producing lymphocyte content in EAE rats [11]. It is important to note that these results were obtained in distinct experimental conditions (experimental models in animals). Nevertheless, the discrepancy of the results suggests the need for further research.

Finally, our study revealed the direct effect of the β_2_-adrenergic receptor agonist and antagonist on IL-17 and IFN-γ production by stimulated CD4^+^ T cells and PBMCs in MS patients and healthy subjects. Stimulation of the β_2_-adrenergic receptor with formoterol suppressed IFN-γ production by CD4^+^ T cells and PBMCs in both groups, while blockade of β_2_-adrenergic receptor did not influence IFN-γ production (Figure 2c,d and Figure 3c,d). This result corresponds to the data in the literature. It has been shown that another selective β_2_-adrenergic receptor agonist, fenoterol, dose-dependently inhibits IFN-γ mRNA expression in the T cells of healthy subjects. The inhibition of IFN-γ expression in this study was blocked by the selective β_2_-adrenergic receptor antagonist ICI 118.551 at the same concentration as in the present study (10^−6^ M), while the selective β_1_-adrenergic receptor antagonist, atenolol, did not have any effect [15].

In contrast, IL-17 production did not change upon β_2_-adrenergic receptor stimulation, while blockade of the β_2_-adrenergic receptor reduced IL-17 secretion by CD4^+^ T cells and PBMCs in both groups (Figure 2a,b and Figure 3a,b). Thus, β_2_-adrenergic receptor stimulation or blocking can have opposite effects on IL-17 and IFN-γ production by CD4^+^ T cells and PBMCs. These results are in line with the data in the literature. In particular, Gonczi et al. showed that the β_2_-adrenergic receptor agonist terbutaline increases IL-17 but suppresses IFN-γ production by stimulated PBMCs and CD4^+^ T cells in healthy subjects, which suggests a reciprocal regulation of Th1 and Th17 cells by the β_2_-adrenergic receptor [16]. The same effect was observed in the study by Barbieri et al., who found a decrease in IL-17A levels and an increase in IFN-γ levels in mice treated with ICI 118.551 [17]. On the other hand, Liu et al. reported the inhibitory effect of the β_2_-adrenergic receptor agonist terbutaline on the shift towards the Th17 phenotype of CD4^+^ T cells in mice with collagen-induced arthritis [14].

Taken together, the results of present and previous studies suggest the bidirectional effect of the β_2_-adrenergic receptor on Th1 and Th17 cells. The mechanism of this effect is still unclear. Signaling through the β_2_-adrenergic receptor increases the level of intracellular cyclic adenosine monophosphate (cAMP) (through activation of adenylate cyclase), which activates downstream targets, such as protein kinase A (PKA) [18]. The role of the cAMP–PKA pathway in the reciprocal regulation of IFN-γ and IL-17 production can be confirmed by the similar effect of dibutyryl cyclic adenosine monophosphate (dBcAMP), a cell-permeable synthetic analog of cAMP [16]. However, in addition to the classical signaling, non-canonical β_2_-adrenergic receptor signaling has also been discussed. Its mechanisms are associated with G-protein-coupled receptor kinases (GRKs). High agonist concentrations can induce GRK-5/6-mediated phosphorylation of the β_2_-adrenergic receptor, leading to the activation of ERK/MAPK signaling (extracellular signal-regulated kinase/mitogen-activated protein kinase) [18]. This type of β_2_-adrenergic receptor signaling might underlie the differential regulation of cytokine production by β_2_-adrenergic receptor agonists.

In addition, the different effects of β_2_-adrenergic receptor blockade/activation on IFN-γ and IL-17 production can depend on the receptor affinity, experimental conditions, and various signaling pathways in T-helper cells. In any case, the β_2_-adrenergic receptor can be considered a promising therapeutic target in CNS autoimmune diseases, which is confirmed by the clinical efficacy of β_2_-adrenergic receptor agonists in EAE and MS courses [19]. However, more studies are needed to clarify the mechanisms underlying the involvement of the β_2_-adrenergic receptor in the modulation of IFN-γ and IL-17 production and their prospects as a new pathogenetic targets in MS.

## 4. Materials and Methods

### 4.1. Patients

Twenty-five patients with a documented diagnosis of MS, according to the McDonald criteria (revised diagnostic criteria for MS, modification 2017), were examined [20]. All patients had a relapsing-remitting form of the disease. All patients were examined during clinical remission. Their main demographic and clinical characteristics are presented in Table 4. All patients were subjected to a standard neurological examination with an assessment of the EDSS score [21]. All patients had been treated with glatiramer acetate for more than one year. At the time of blood sampling, all patients in the study had not been treated with corticosteroid therapy and selective serotonin or serotonin-norepinephrine reuptake inhibitors for more than six months. All patients were non-smoking and had no mental disorders, according to the Beck Depression Inventory, and no cognitive impairments, according to Montreal Cognitive Assessment [22,23]. The control group consisted of sixteen healthy donors matched with patients by sex and age (Table 4).

### 4.2. Epinephrine, Norepinephrine, and 3-Methoxy-4-Hydroxyphenylglycol Evaluation

The concentration of epinephrine, norepinephrine, and its metabolite MHPG in blood plasma were determined by high-performance liquid chromatography (HPLC), as previously described [24]. Epinephrine, norepinephrine, and MHPG concentrations in samples were calculated by the “internal standard” method, based on the ratio of peak areas in the standard mixture and in the sample, and expressed in pmol/mL.

### 4.3. Peripheral Blood Mononuclear Cells and CD4^+^ T Cell Cultures and Stimulation

The functional activity of Th1 and Th17 cells was determined, as previously described [25]. Briefly, peripheral blood mononuclear cells (PBMCs) were isolated from whole blood by centrifugation against a Ficoll density gradient. CD4^+^ T cells were isolated from PBMCs by magnetic cell sorting (MACS) using a negative CD4^+^ T cell isolation kit, according to the manufacturer’s instructions (Miltenyi Biotec, Bergisch Gladbach, Germany). CD4^+^ T cells or PBMCs, at a concentration of 8 × 10^4^ per 200 μL per well, were placed in 96-well U-bottomed culture plates (SPL, Pocheon, Korea) in duplicates and stimulated with microbeads coated with anti-CD3 and anti-CD28-antibodies (Life Technologies, Oslo, Norway) for 72 h in a CO_2_-incubator, whereafter the culture supernatants were collected and stored at −70 °C [26]. Negative control samples were cultured without stimulation.

To assesses the effect of norepinephrine on the function of Th1 and Th17 cells, CD4^+^ T cells were cultured in the presence of norepinephrine (Sigma, St. Louis, MO, USA) at concentrations of 10^−6^ M, 10^−5^ M, and 10^−4^ M, whereafter anti-CD3/anti-CD28 microbeads were added to the cultures and the stimulation proceeded, as described above [10].

To study the involvement of the β_2_-adrenergic receptor in the norepinephrine-mediated modulation of cytokine production, some samples of CD4^+^ T cells or PBMCs were pre-incubated with the β_2_-adrenergic receptor antagonist ICI 118.551 or the agonist formoterol at a concentration of 10^−6^ M (both from Tocris, Bristol, UK), whereafter norepinephrine (10^−5^ M) and anti-CD3/anti-CD28 microbeads were added to the cultures [27,28]. In addition, to study the direct effect of the β_2_-adrenergic receptor antagonist and agonist on cytokine production, the samples of CD4^+^ T cells or PBMCs were pre-incubated with ICI 118.551 or formoterol, respectively, and activated by anti-CD3 and anti-CD28 microbeads.

### 4.4. Cytokine Evaluation

Levels of IL-17 and IFN-γ in the culture supernatants were determined by ELISA (Invitrogen, Carlsbad, CA, USA). The lower limit of detection was 4 pg/mL for both cytokines. In all cases of ELISAs, the instructions of the kit manufacturers were followed. The data are expressed as pg/mL or as the percentage of cytokine production by stimulated CD4^+^ T cells in the absence of norepinephrine and antagonist or agonist of the β_2_-adrenoreceptor.

### 4.5. Statistical Analysis

The statistical analysis of the results was performed using Prizm 6 software. To compare two groups the nonparametric Mann–Whitney U-test or Wilcoxon signed-rank test were used. One-way ANOVA, followed by the Bonferroni correction, was used for multiple comparisons. Differences were considered statistically significant if *p* < 0.05.

## Figures and Tables

**Table 1:** The secretion of cytokines by CD4^+^ T cells (ELISA) in MS patients and in healthy subjects. Data are medians (25th and 75th percentiles).

Factor	Stimulation	MS Patients, *n* = 25	Healthy Subjects, *n* = 16
IL-17, pg/mL	None	0 (0; 3)	4 (2; 8)
	Anti-CD3/CD28	503 (237; 1098)	632 (251; 874)
IFN-γ, pg/mL	None	7 (3; 24)	2 (0; 26)
	Anti-CD3/CD28	5598 (3987; 7491)	4991 (2803; 5846)

**Table 2:** The secretion of cytokines by PBMCs (ELISA) in MS patients and in healthy subjects. Data are medians (25th and 75th percentiles).

Factor	Stimulation	MS Patients, *n* = 15	Healthy Subjects, *n* = 15
IL-17, pg/mL	None	0 (0; 2)	0 (0; 1)
	Anti-CD3/CD28	325 (171; 498)	301 (184; 495)
IFN-γ, pg/mL	None	7 (4; 23)	7 (3; 9)
	Anti-CD3/CD28	2790 (2232; 5232)	2409 (1213; 5439)

**Table 3:** The concentration of epinephrine, norepinephrine, and its metabolite MHPG in blood plasma (HPLC) in MS patients and in healthy subjects. Data are medians (25th and 75th percentiles).

Factor	MS Patients, *n* = 25	Healthy Subjects, *n* = 16
Epinephrine, pmol/mL	36.97 (21.3; 78.16)	38.22 (29.9; 64.02)
Norepinephrine, pmol/mL	1772 (1317; 2262)	1733 (1153; 2564)
MHPG, pmol/mL	262.9 (139.7; 392.7)	214.2 (148.6; 282.5)

**Table 4:** Clinical and demographic characteristics of MS patients and healthy subjects. Data are medians (25th and 75th percentiles).

Factor	MS Patients, *n* = 25	Healthy Subjects, *n* = 16
Age, years	31 (25; 34)	30 (28; 33)
Men/women (% women)	8/17 (68)	6/10 (63)
Duration of MS, years	2 (2; 5)	NA
EDSS, score	1.5 (1.5; 2)	NA

NA: not applicable.

## Data Availability

The data presented in this study are available on request from the corresponding author.
